# Microscopic polyangiitis associated with thymic tumor: a case report and review of the literature

**DOI:** 10.1186/s12882-019-1319-9

**Published:** 2019-04-08

**Authors:** Yasunori Miyamoto, Kouichi Hirayama, Hiroshi Maruyama, Kentaro Ohgi, Mamiko Takayasu, Homare Shimohata, Masaki Kobayashi

**Affiliations:** 1 Department of Nephrology, Tokyo Medical University Ibaraki Medical Center, 3-20-1 Chuo, Ami, Ibaraki 300-0395 Japan; 2 Department of Internal Medicine, Miyamoto Hospital, Inashiki, Ibaraki Japan

**Keywords:** Microscopic polyangiitis, MPO- ANCA, PR3-ANCA, Azurocidin-ANCA, Thymoma

## Abstract

**Background:**

Thymic hyperplasia and thymic epithelial tumor (thymoma) have been associated with a variety of autoimmune diseases. Renal involvement has been reported in patients with thymoma. Minimal change disease and membranous nephropathy are frequently observed in glomerular lesions of thymoma patients, but ANCA-associated renal vasculitis is rare. We present a case of thymoma-associated microscopic polyangiitis with positivity for three ANCAs: MPO-ANCA, PR3-ANCA and azurocidin-ANCA.

**Case presentation:**

An 89-year-old Japanese woman was admitted to our hospital following an episode of general fatigue, nausea, muscle weakness of the lower limbs, and ophthalmoplegia. On urinalysis, proteinuria, hematuria, and cellular casts were observed. Elevated levels of serum creatinine and C-reactive protein were also demonstrated, and MPO-, PR3- and azurocidin-ANCA were detected on serological examination. Renal biopsy showed pauci-immune crescentic glomerulonephritis. We therefore diagnosed rapidly progressive glomerulonephritis due to microscopic polyangiitis. Acetylcholine-receptor antibody was also detected. Chest computed tomography and MRI revealed a lobulated tumor in the anterior mediastinum. We thus also diagnosed myasthenia gravis with thymoma.

**Conclusion:**

Considering the patient’s triple-ANCA positivity, thymic diseases may be associated with the pathogenesis of ANCA-associated vasculitis due to central T-cell tolerance. A further accumulation of cases is needed, because thymectomy does not always induce the remission of thymoma-associated autoimmune diseases.

**Electronic supplementary material:**

The online version of this article (10.1186/s12882-019-1319-9) contains supplementary material, which is available to authorized users.

## Background

Thymic hyperplasia and thymic epithelial tumor (thymoma) have been associated with a variety of autoimmune diseases [1–3]. The authors of a review of 598 patients with thymoma reported that 71% of those patients had several types of immune-mediated disorders, and the most prevalent disorders were myasthenia gravis followed by pure red cell aplasia, polymyositis, and systemic lupus erythematosus [1]. A high frequency of autoimmune diseases in patients with thymic tumor and the efficacy of thymectomy for achieving the remission of autoimmune diseases were also reported [2].

Renal disease has rarely been observed in association with thymoma. An association of glomerulonephritis with thymic tumor was first described in 1980 [4]. That case report was of a 48-year-old male who presented with nephrotic syndrome with thymoma; a renal biopsy revealed membranous glomerulonephritis. In a retrospective study of renal diseases with thymoma, minimal change nephrotic disease was frequently seen on glomerular lesions [5].

Although thymic disease is associated with various autoimmune diseases, ANCA-associated vasculitis has rarely been observed [5–10]. We herein describe a case of ANCA-associated vasculitis with thymoma, and we discuss the characteristics of such patients with reference to previous reports.

## Case presentation

An 89-year-old Japanese woman was admitted to our hospital because of general fatigue, nausea, muscle weakness of the lower limbs, and ophthalmoplegia that had appeared 2 months earlier and gradually worsened. She noticed diplopia at 1 year before this hospitalization. None of her family members had a history of renal or autoimmune disease. There was no past history of allergic diseases such as bronchial asthma. The patient had never smoked. She had been diagnosed with hypertension and treated with an angiotensin-receptor antagonist (losartan, 50 mg daily).

The physical examination identified a slight fever, anemic conjunctiva, eyelid ptosis, and muscle weakness of the lower limbs; her blood pressure was 135/67 mmHg. The laboratory examination revealed elevated serum levels of creatinine (1.91 mg/dL, 170 μmol/L) and C-reactive protein (8.12 mg/dL). A hemogram showed anemia (hemoglobin concentration, 9.2 g/dL), but her white blood cell count was in the normal range (5400 /μL). On urinalysis, occult blood and protein and cellular (granular and red blood cell) casts were detected. Her urinary protein excretion was 2.13 g/g creatinine. The serological examination revealed an elevated level of serum IgG (3494 mg/dL), but serum complement levels were within normal limits: the serum C_3_ level was 95.1 mg/dL, the serum C_4_ level was 21.7 mg/dL, and the serum CH_50_ level was 44.7 U/mL. Positivity for MPO-ANCA (176 U/mL) and PR3-ANCA (125 U/mL) was observed, but rheumatoid factor, antinuclear antibody, anti-glomerular basement membrane antibody, cryoglobulin, and monoclonal immunoglobulin were not detected.

We also assayed for various types of ANCA in the patient’s serum. A sample was collected in a separator tube before the patient’s initial treatment and was separated at 1000 *g* for 15 min. The diluted serum sample was measured by an enzyme-linked immunosorbent assay (ELISA) using a Wieslab^®^ ANCA panel kit (EuroDiagnostica, Malmo, Sweden), in duplicate. The ELISA plate was read on a microplate reader (Sunrise Remote^®^: Tecan Japan, Kanagawa, Japan) set at 405 nm wavelength. The patient showed positivity for azurocidin-ANCA (optical density [OD] ratio: 4.05, normal: < 3.0), but not bactericidal/permeability increasing protein (BPI)-ANCA (OD ratio: 1.61, normal: < 3.0), cathepsin G-ANCA (OD ratio: 1.20, normal: < 3.0), elastase-ANCA (OD ratio: 0.99, normal: < 3.0), lactoferrin-ANCA (OD ratio: 2.77, normal: < 3.0) or lysozyme-ANCA (OD ratio: 1.47, normal: < 3.0) [see Additional file 1].

**Fig. 1:**
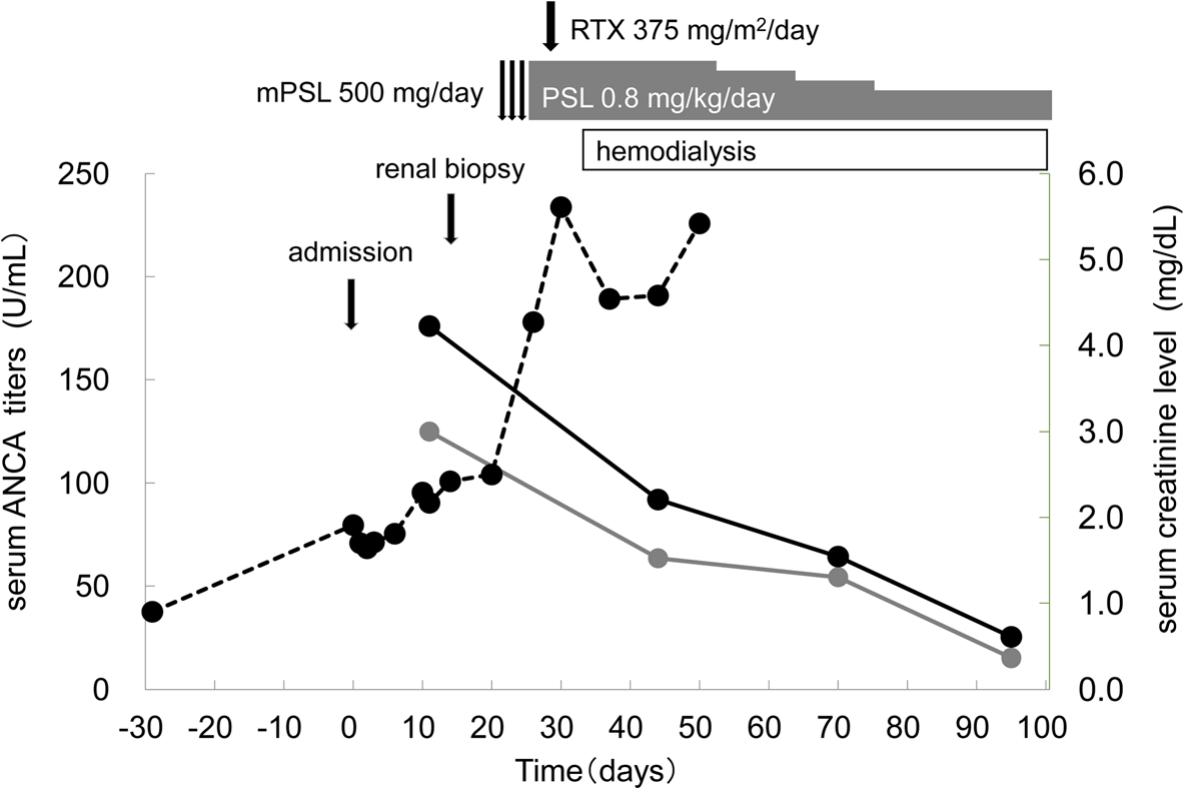
Clinical course of the present case. mPSL, methylprednisolone; PSL, prednisolone; RTX, rituximab. *Solid black line:* Serum MPO-ANCA levels. *Solid gray line:* Serum PR3-ANCA levels. *Dotted black line:* Serum creatinine levels

The patient’s symptoms and inflammatory findings did not improve with antibiotic treatment (ceftriaxone, 2 g daily for 6 days), and her serum creatinine level deteriorated to 2.42 mg/dL (Fig. 1). On abdominal ultrasound examination, her kidney size was relative small (right, 78 mm × 40 mm; left, 87 mm × 46 mm). We diagnosed rapidly progressive glomerulonephritis.

**Fig. 2:**
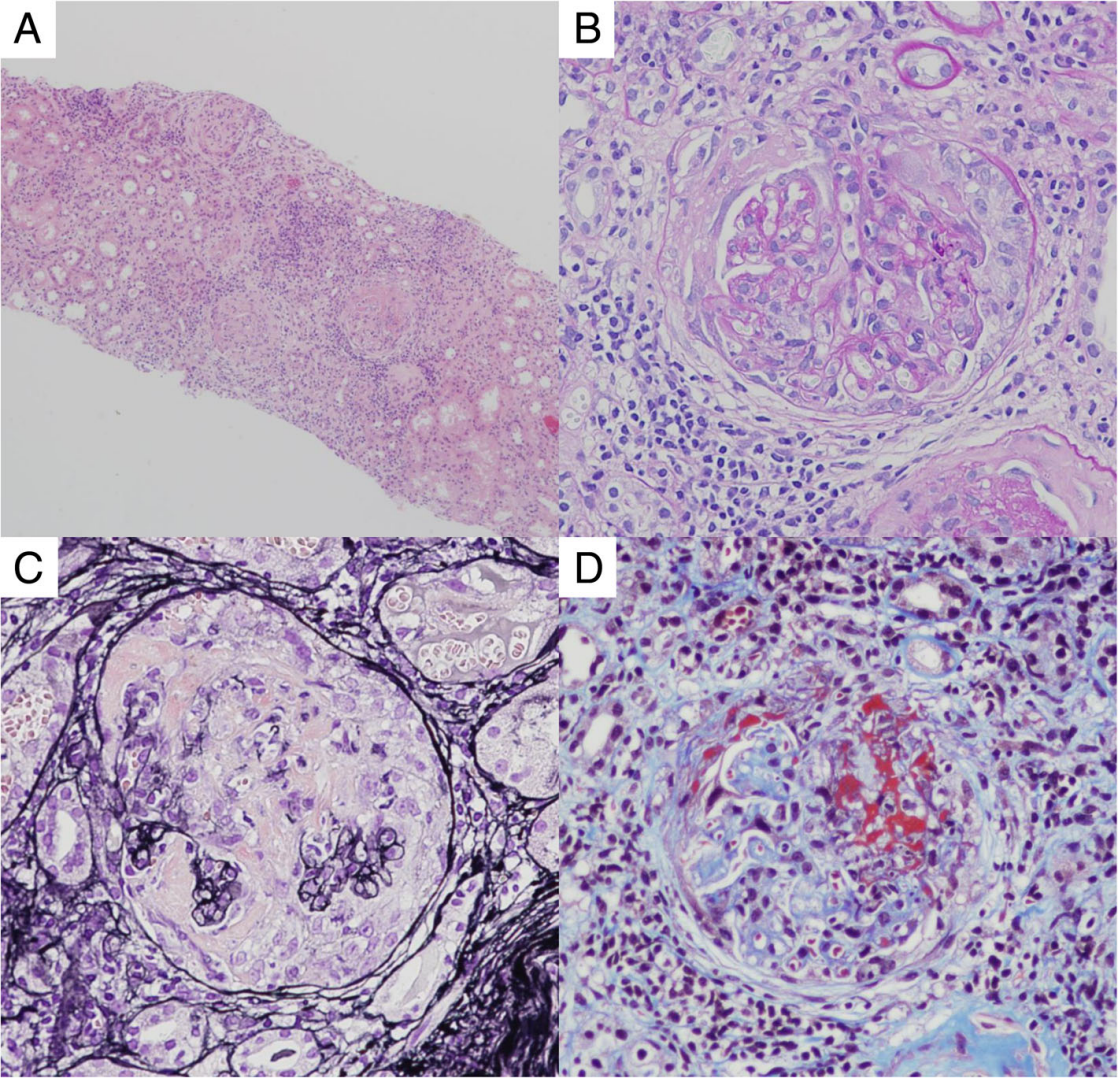
Renal histopathological findings in the present case. **a** Hematoxylin-eosin staining (40×). **b** Periodic acid-Schiff staining (200×). **c** Periodic acid-methenamine silver staining (400×). **d** Masson’s Trichrome staining (400×)

Light microscopic findings of a renal biopsy sample showed cellular crescents in 50% of 14 obtained glomeruli, and a fibrocellular crescent was revealed in one of those glomeruli. Mononuclear inflammatory cell infiltration to the interstitium was widely observed. Vasculitis was not observed, but intimal thickening of the interlobular arterial walls was seen (Fig. 2). On immunofluorescence findings, immunoglobulins and complement components were not detected. We therefore diagnosed microscopic polyangiitis with cellular-type renal involvement.

**Fig. 3:**
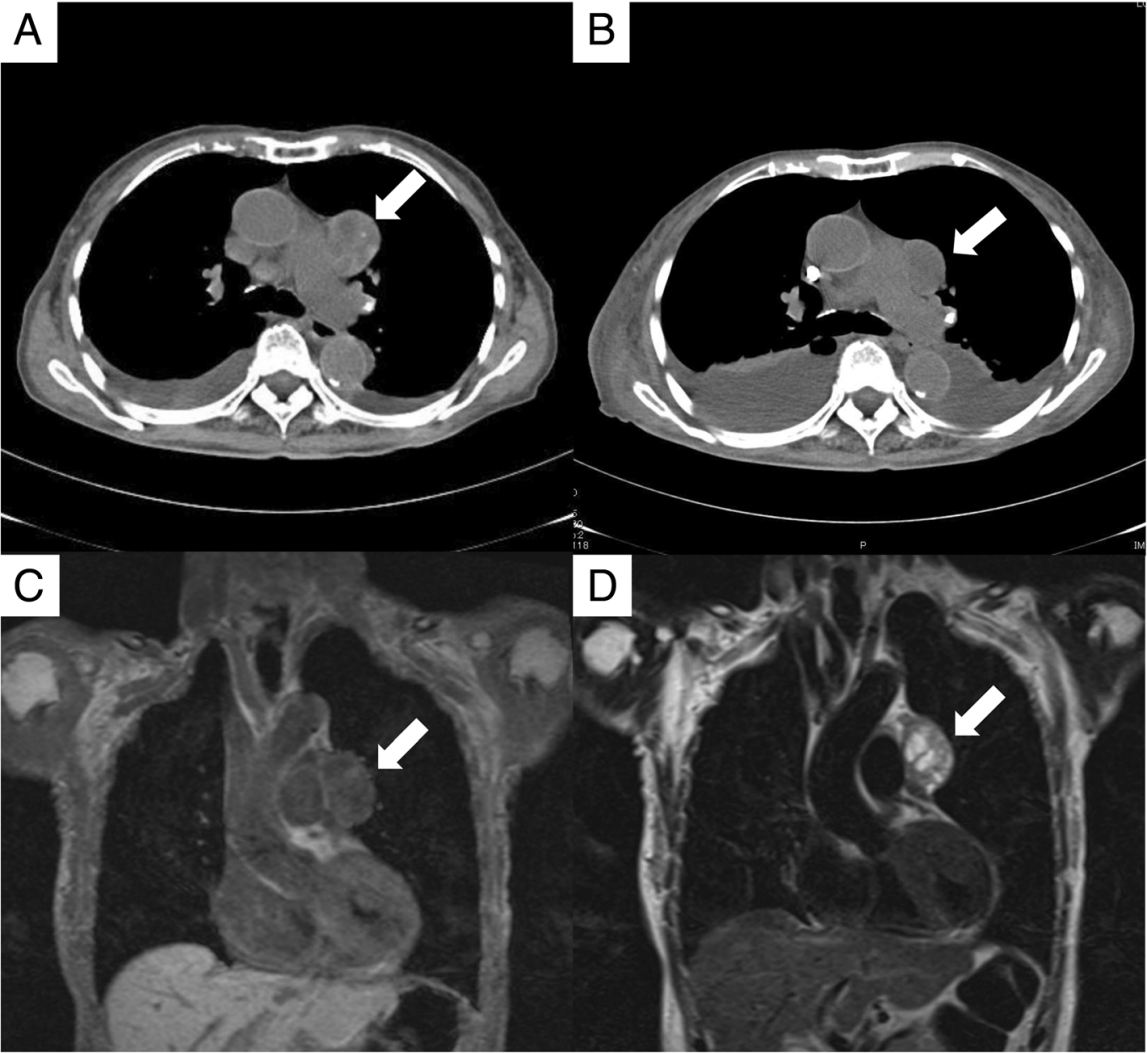
Findings of radiological examinations in the present case. **a** Chest CT. **b** Chest CT 2 months after the treatment. **c** Chest MRI, T1-weighted imaging coronal section. **d** Chest MRI T2-weighted imaging coronal section. *Arrows* indicate the lobulated tumor in the anterior mediastinum

Chest X-rays showed a wide mediastinum, and chest computed tomography (CT) and magnetic resonance imaging (MRI) revealed a 40-mm-sized lobulated tumor in the anterior mediastinum (Fig. 3). On additional serological examination, anti-acetylcholine-receptor antibody was present (0.9 nmol/L), but anti-muscle specific kinase (MuSK) antibody was not detected. We thus additionally diagnosed myasthenia gravis with thymoma.

After the renal biopsy, the patient was treated with 500 mg/day of methylprednisolone for three consecutive days, followed by 30 mg/day (0.8 mg/kg) of oral prednisolone. Rituximab 500 mg (375 mg/m^2^) was intravenously administered, but it was discontinued after only one administration because of an adverse effect (atrial fibrillation). Three months after the remission induction therapy, the patient’s MPO-ANCA was decreased to 25.3 U/mL, PR3-ANCA was decreased to 15.2 U/mL, and elevated serum IgG level was normalized to 1297 mg/dL. Her symptoms had improved; at 2 months after the initiation of treatment, anti-acetylcholine-receptor antibody became negative and the size of her thymoma had decreased to approx. 20 mm (Fig. 3). However, hemodialysis was initiated at 1 month after this treatment because the patient’s renal function was deteriorated. Her renal function did not unfortunately recovered, and she was moved to another hospital 3 months after the therapy because she needed rehabilitation for her muscle weakness due to disuse syndrome.

## Discussion and conclusion

ANCA-associated vasculitis has rarely been observed in association with thymoma; we found only seven cases reported previously (Table 1) [5–10]. In four cases, pauci-immune extracapillary glomerulonephritis was observed, similar to our patient’s case. Myasthenia gravis was complicated in our patient, and six of the seven reported patients also had other autoimmune diseases, such as myasthenia gravis and pure red cell aplasia. Two of the seven cases were preceded by thymoma; both diseases were simultaneously present in three cases, and preceding vasculitis occurred in two cases.

**Table 1:** Characteristics of ANCA-associated vasculitis complicated with thymoma (review)

No.	authors	year	age sex	vasculitis		ANCA	other associated diseases	preced. diseases	laboratory data			renal pathology	
				class	organs				UP (g/d)	s-Alb (g/L)	s-Cr (mg/dL)	findings	class
1	Haberhauer G, et al.	1993	22 F	NC	skin	perinuclear	PRCA	thymoma	NA	NA	NA	LC vasc. of skin	
2	Kobayashi M, et al.	1995	47 F	absent		perinuclear	ITP, MG	same	NA	NA	NA	no vasculitis	
3	Valli G, et al.	1998	70 M	NC	kidney	NA	MG	thymoma	3.9	NA	1.6 → 11.4	ECPGN	mixed
4	Karras A, et al.	2005	35 F	NC	kidney	NA	MG, PRCA	vasculitis	1.8	NA	13.5	ECPGN	NA
5	Karras A, et al.	2005	65 M	NC	kidney	NA	MG	same	1.0	35	1.69	ECPGN	NA
6	Parambil JG, et al.	2006	50 M	MPA	kidney	MPO	none	same^a^	3.9	NA	3.5	ECPGN	NA
7	Holmes MV, et al.	2007	7 F	MPA	kidney	negative	MG	same vasculitis	NA	NA	NA	ECPGN	NA
8	the present case	2018	89 F	MPA	kidney	MPO, PR3, azurocidin	MG	same	2.1	29	2.17	ECPGN	cres.

*F* female, *M* male, *NC* not classified, *ND* not diagnosed, *NA* not available, *EU* ELISA units, *PRCA* pure red cell aplasia, *ITP* idiopathic thrombocytopenic purpura, *MG* myasthenia gravis, *Preced* precedence, *UP* urinary protein, *S-Alb* serum albumin; S-Cr serum creatinine; NA not available, LC vasc, leucocytoclastic vasculitis, ECPGN extracapillary proliferative glomerulonephritis; cres, crescentic

^a^Vasculitis was exacerbated after thymectomy

Although three of the seven reported patients demonstrated perinuclear-ANCA or MPO-ANCA, in our patient’s case triple-ANCA (MPO, PR3 and azurocidin-ANCA) was detected. The mechanism of ANCA production has been unclear, but it may involve failures of self-immune tolerance mechanisms. Self-immune tolerance is divided into two categories: central tolerance and peripheral tolerance. Multiple factors in the immune system are critical for the establishment of self-immune tolerance [11]. Regulatory T cells, which efficiently suppress autoreactive T-cell responses in vitro and in vivo, may be major contributors to peripheral tolerance [12]. In ANCA-associated vasculitis, abnormalities in the number and function of regulatory T cells have been observed [13–15], and peripheral immune tolerance abnormality in regulatory T cells is one of the pathogeneses of ANCA-associated vasculitis.

In contrast, thymoma-associated autoimmune diseases may be caused by abnormalities of central immune tolerance due to the induction of autoreactive T-cell clones in abnormal thymic tissue or to the suppression of regulatory T cells [16]. Considering the complication of thymoma and the detection of multiple ANCAs (MPO-, PR3-, and azurocidin-ANCA) in our patient, an abnormality of central immune tolerance may also be one of the pathogeneses of ANCA-associated vasculitis.

In a patient with invasive thymoma, extracapillary proliferative glomerulonephritis was observed, but ANCA was not identified (Tables 1 and 2). Among the three reported patients with noninvasive thymoma, extracapillary proliferative glomerulonephritis with ANCA was observed in two patients and ANCA was not available in the other. B-lymphocyte infiltration was observed in one patient, but detailed findings of B-lymphocyte infiltration to the thymus were not available in the other five patients. However, we could not identify the association between ANCA positivity and histological findings of the thymus in our literature review because of the small number of patients. A further accumulation of ANCA-associated vasculitis patients with thymoma is needed.

**Table 2:** Treatments of ANCA-associated vasculitis complicated with thymoma (review)

No.	authors	year	thymectomy	thymic pathology		medication for vasculitis			outcome	
				findings	class	CS	CYC	others	remission	relapse
1	Haberhauer G, et al.	1993	done	hyperplasia		+	–	NSAID	CR	relapse
2	Kobayashi M, et al.	1995	done	non-invasive	B2	30 mg/d	–	G-CSF^a^	CR^a^	none
3	Valli G, et al.	1998	done	NA	NA	+	+	–	ESRD	NA
4	Karras A, et al.	2005	done	non-invasive	B2	+	–	AZA	CR	NA
5	Karras A, et al.	2005	not done			+	–	–	PR	NA
6	Parambil JG, et al.	2006	done	non-invasive	B2	1.0 mg/kg/d	2.0 mg/kg/d	AZA	CR	none
7	Holmes MV, et al.	2007	done	invasive	B1	+	+	–	CR	NA
8	The present case	2018	not done			1.0 mg/kg/d	–	Rituximab	ESRD	none

*CS* corticosteroid, *CYC* cyclophosphamide, *NSAID* non-steroidal anti-inflammatory drugs, *G-CSF* granulocyte-colony stimulating factor, *AZA* azathioprine, *CR* complete remission, *PR* partial remission, *ESRD* end-stage renal disease, *NA* not available

^a^Medications and prognosis for granulocytopenia

A thymectomy may be useful to clarify the association between a patient’s thymoma and ANCA production, but in our patient’s case a thymectomy could not be performed because of her advanced age. Moreover, a thymectomy does not always induce the clinical and immunologic remission of associated autoimmune syndromes, and in some cases it causes disease progression or the occurrence of an autoimmune disease [9]. In our survey of the literature (Table 2), there were six cases in which a thymectomy was performed, and it did not always induce the remission of the patients’ disorders. The remission of vasculitis was achieved in only one patient after thymectomy; flares of vasculitis were observed in two patients, a relapse occurred in one patient, and there was no change of vasculitis activity in one patient. We decided not to perform a thymectomy for our patient due to her age but also considering that vasculitis relapsed or was exacerbated after thymectomy in several past cases.

Although MPO and PR3 are major antigens of ANCA, several other antigens have been recognized, including azurocidin. In a study of 185 patients with ANCA by immunofluorescence examination, 20 patients had azurocidin-ANCA and 13 of those 20 patients revealed renal vasculitis [17]. Azurocidin-ANCA may therefore be related to necrotizing crescentic glomerulonephritis. On the other hand, another study of 376 ANCA-positive patients who underwent a renal histological examination, azurocidin-ANCA was detected in 19 of 229 patients with perinuclear-ANCA and three of 99 patients with cytoplasmic-ANCA [18]. However, in that study, only three of 168 (1.8%) patients with necrotizing crescentic glomerulonephritis had azurocidin-ANCA, but azurocidin-ANCA was detected in 19 of 160 (11.9%) patients with other glomerular diseases [19]. Therefore, azurocidin-ANCA may not be specific to necrotizing crescentic glomerulonephritis.

The pathogenicity of azurocidin-ANCA has not been established. In our patient’s case, disturbed central T-cell tolerance due to the thymoma may have induced the triple ANCA, but azurocidin-ANCA may not be pathogenetic since her azurocidin-ANCA titer was relative low compared to those of MPO- and PR3-ANCA. A further accumulation of patients with azurocidin-ANCA-associated renal vasculitis is needed to clarify the clinical and pathologic roles of azurocidin-ANCA.

Based on the positivity of MPO-, PR3-, and azurocidin-ANCA in our patient’s case, thymic diseases might be associated with the pathogenesis of ANCA-associated vasculitis due to central T-cell tolerance. Achieving the remission of polyangiitis by performing a thymectomy is necessary to clarify the mechanism mentioned above. However, a new onset and/or exacerbation of vasculitis due to thymectomy have also been reported. To establish a treatment for thymoma-associated autoimmune diseases, further case studies are necessary.

## Additional file

Additional file 1: ELISA data for various types of ANCA. The patient sample is negative for azurocidine, elastase, lactoferrin and lysozome if the optical density (OD) value is < 0.4. The patient sample is negative for BPI and cathepsin G if the OD value is < 0.6. If the patient OD value is > 0.4 on azurocidine, elastase, lactoferrin or lysozome, or if the patient OD value is > 0.6 on BPI or cathepsin G, an OD ratio for the antigen is calculated as follows: the OD ratio = (OD of patients sample for the antigen) / (OD of patients sample blank). The patient sample is negative if the OD ratio is < 3.0 and positive if the OD ratio is > 4.0. For the quality of analysis, the OD value for the patients sample in the blank should be < 0.35 and the OD value for the assay control (human IgG) should be > 1.0. (Referred the manual of a Wieslab^®^ ANCA panel kit (EuroDiagnostica, Malmo, Sweden)) (DOCX 14 kb)

## Abbreviations

ANCA: Antineutrophil cytoplasmic antibody
BPI: Bactericidal permeability increasing protein
ELISA: Enzyme-linked immunosorbent assay
MPO: Myeloperoxidase
OD: Optical density
PR3: Proteinase 3
