# Supplementary material for: Microscopic polyangiitis associated with thymic tumor: a case report and review of the literature
Source: BMC Nephrol. 2019 Apr 8;20:123. doi: 10.1186/s12882-019-1319-9 (PMC6454690; doi:10.1186/s12882-019-1319-9)
Supplement: Supplementary file 1 — ELISA data for various types of ANCA. The patient sample is negative for azurocidine, elastase, lactoferrin and lysozome if the optical density (OD) value is < 0.4. The patient sample is negative for BPI and cathepsin G if the OD value is < 0.6. If the patient OD value is > 0.4 on azurocidine, elastase, lactoferrin or lysozome, or if the patient OD value is > 0.6 on BPI or cathepsin G, an OD ratio for the antigen is calculated as follows: the OD ratio = (OD of patients sample for the antigen) / (OD of patients sample blank). The patient sample is negative if the OD ratio is < 3.0 and positive if the OD ratio is > 4.0. For the quality of analysis, the OD value for the patients sample in the blank should be < 0.35 and the OD value for the assay control (human IgG) should be > 1.0. (Referred the manual of a Wieslab® ANCA panel kit (EuroDiagnostica, Malmo, Sweden)) (DOCX 14 kb) [file 12882_2019_1319_MOESM1_ESM.docx]

**Additional file 1. ELISA data for various types of ANCA**

| ANCA  types | OD  1 | OD  2 | mean  OD | OD ratio 1 | OD ratio 2 | OD  ratio  mean | negative OD  range | negative ratio  range | positive ratio  range | quality control (OD) | CV-  value  (%) |
| --- | --- | --- | --- | --- | --- | --- | --- | --- | --- | --- | --- |
| blank | 0.2619 | 0.2649 | 0.2634 | － | － | － | － | － | － | < 0.35 | 0.81 |
| azurocidin | 1.2009 | 1.1299 | 1.1654 | 4.5853 | 4.2654 | 4.4254 | < 0.4 | < 3.0 | > 4.0 | － | 4.31 |
| BPI | 0.4169 | 0.4309 | 0.4239 | 1.5918 | 1.6267 | 1.6092 | < 0.6 | < 3.0 | > 4.0 | － | 2.34 |
| cathepsin G | 0.3129 | 0.3209 | 0.3169 | 1.1947 | 1.2114 | 1.2031 | < 0.6 | < 3.0 | > 4.0 | － | 1.79 |
| elastase | 0.2549 | 0.2659 | 0.2604 | 0.9733 | 1.0038 | 0.9885 | < 0.4 | < 3.0 | > 4.0 | － | 2.99 |
| lactoferrin | 0.7499 | 0.7069 | 0.7284 | 2.8633 | 2.6686 | 2.7659 | < 0.4 | < 3.0 | > 4.0 | － | 4.17 |
| lysozyme | 0.3739 | 0.4019 | 0.3879 | 1.4276 | 1.5172 | 1.4724 | < 0.4 | < 3.0 | > 4.0 | － | 5.10 |
| human IgG | 2.8479 | 3.0889 | 2.9684 | 10.8740 | 11.6606 | 11.2673 | － | － | － | > 1.0 | 5.74 |

The patient sample is negative for azurocidine, elastase, lactoferrin and lysozome if the optical density (OD) value is < 0.4. The patient sample is negative for BPI and cathepsin G if the OD value is < 0.6. If the patient OD value is >0.4 on azurocidine, elastase, lactoferrin or lysozome, or if the patient OD value is > 0.6 on BPI or cathepsin G, an OD ratio for the antigen is calculated as follows: the OD ratio = (OD of patients sample for the antigen) / (OD of patients sample blank). The patient sample is negative if the OD ratio is < 3.0 and positive if the OD ratio is > 4.0.

For the quality of analysis, the OD value for the patients sample in the blank should be < 0.35 and the OD value for the assay control (human IgG) should be > 1.0.

(Referred the manual of a Wieslab^®^ ANCA panel kit (EuroDiagnostica, Malmo, Sweden))
